# Large-scale genomic analysis of *Mycobacterium tuberculosis* reveals extent of target and compensatory mutations linked to multi-drug resistant tuberculosis

**DOI:** 10.1038/s41598-023-27516-4

**Published:** 2023-01-12

**Authors:** Gary Napier, Susana Campino, Jody E. Phelan, Taane G. Clark

**Affiliations:** 1 Department of Infection Biology, Faculty of Infectious and Tropical Diseases, London School of Hygiene & Tropical Medicine, London, WC1E 7HT UK; 2 Faculty of Epidemiology and Population Health, London School of Hygiene & Tropical Medicine, London, WC1E 7HT UK

**Keywords:** Genome informatics, Microbial genetics

## Abstract

Resistance to isoniazid (INH) and rifampicin (RIF) first-line drugs in *Mycobacterium tuberculosis* (Mtb), together called multi-drug resistance, threatens tuberculosis control. Resistance mutations in *katG* (for INH) and *rpoB* (RIF) genes often come with fitness costs. To overcome these costs, Mtb compensatory mutations have arisen in *rpoC*/*rpoA* (RIF) and *ahpC* (INH) loci. By leveraging the presence of known compensatory mutations, we aimed to detect novel resistance mutations occurring in INH and RIF target genes. Across ~ 32 k Mtb isolates with whole genome sequencing (WGS) data, there were 6262 (35.7%) with INH and 5435 (30.7%) with RIF phenotypic resistance. Known mutations in *katG* and *rpoB* explained ~ 99% of resistance. However, 188 (0.6%) isolates had *ahpC* compensatory mutations with no known resistance mutations in *katG*, leading to the identification of 31 putative resistance mutations in *katG,* each observed in at least 3 isolates. These putative *katG* mutations can co-occur with other INH variants (e.g., *katG*-Ser315Thr, *fabG1* mutations). For RIF, there were no isolates with *rpoC*/*rpoA* compensatory mutations and unknown resistance mutations. Overall, using WGS data we identified putative resistance markers for INH that could be used for genotypic drug-resistance profiling. Establishing the complete repertoire of Mtb resistance mutations will assist the clinical management of tuberculosis.

## Introduction

Tuberculosis (TB), caused by *Mycobacterium tuberculosis* (Mtb) bacteria, is a major global public health problem. TB control is complicated by drug resistance, especially to first-line rifampicin (RIF) and isoniazid (INH), together called multi-drug resistance (MDR-TB). To acquire resistance to anti-TB drugs, Mtb drug targets or activating proteins are often mutated^1^. As a consequence, the biological function of these proteins is impaired or sometimes completely lost^2^, causing the bacterium to incur a fitness cost. These costs can manifest as a phenotypic difference, such as reduced virulence or transmissibility. For example, the *katG* gene codes for the KatG enzyme, a catalase-peroxidase that protects the bacterium from reactive oxygen species damage and is used to detoxify hydrogen peroxide^3^, improving survival within macrophages and the host immune response. The enzyme also activates the pro-drug INH, converting it to an active form^4^.

Mutations in the *katG* gene that disrupt INH binding to KatG often leave Mtb drug resistant and a protein with impaired enzymatic function. In some cases, mutations can confer drug resistance without a punitive fitness cost. For example, the *katG* Ser315Thr mutation confers resistance but minimally affects fitness, hence is highly prevalent among (pre-)MDR-TB strains^5,6^. For RIF, the target is the $\beta$’ subunit of RNA polymerase, coded by the *rpoB* gene. Mutations in *rpoB* prevent RIF from binding, but incur a high fitness cost since the intricate machinery of RNA polymerase is intolerant to large structural changes^7^. One notable exception is *rpoB* Ser450Leu, which is highly prevalent in RIF-resistant strains^8^. Indeed, so restrictive are changes to the $\beta$ subunit, more than 95% of drug resistance mutations occur in the RIF resistance determining region (RRDR), an 81 base-pair section of the *rpoB* gene^9^.

To overcome these fitness costs, secondary mutations can arise that improve or promote either the target protein itself or an alternative with a similar function. In the case of INH/*katG*, the expression of *ahpC*, which codes for a protein with similar enzymatic function, is often increased via mutations in the promoter of the *ahpC* gene^10,11^. RIF compensatory mutations occur in RNA polymerase subunits $\alpha$ (*rpoA*), $\beta$’ (*rpoC*) or even within the $\beta$ subunit (*rpoB*) itself. These mutations are thought to occur at the interfaces of the subunits, helping to restore overall RNA polymerase function, while maintaining an altered binding site in the $\beta$ subunit^12^.

The TB-Profiler platform^6^ uses 2,300 mutations across 35 loci to profile Mtb resistance for 21 anti-TB drugs, including RIF and INH. However, the full repertoire of resistance mutations, including for MDR-TB is not fully characterised. The accompanying TB-Profiler database consists of ~ 32 k isolates with whole genome sequence and drug susceptibility test (DST) phenotypic data, with inferred genotypic profiles. Here, by investigating those isolates with compensatory mutations but no known resistance mutations, we aim to identify the presence of novel mutations linked to genes for INH, RIF, and therefore MDR-TB. Further, we attempt to understand the patterns of co-existence between resistance and compensatory mutations in relation to INH and RIF drug resistance.

## Results

### Isolate data

A total of 32,669 Mtb isolates with whole genome sequencing and DST data were analysed, and encompassed all major lineages (L4 51.1%, L2 25.3%, L3 11.5%, L1 9.7%) (Table 1). Across the 17,524 samples with DST data, 6262 (35.7%), 5435 (30.7%) and 5011 (28.6%) were phenotypically resistant to INH, RIF, and MDR-TB, respectively. Genotypic resistance prediction using TB-Profiler software inferred that 9546 (/32,669; 29.2%) and 7974 (24.4%), 5385 (16.5%) were resistant to INH, RIF, and MDR-TB, respectively (Table 1). The most common mutations underlying INH resistance were *katG* Ser315Thr (n = 7165; 21.9%), *fabG1* -15C>T (n = 1989; 6.1%), and *inhA* -154G>A (n = 332; 1.0%). Similarly, for the RIF resistance, the most frequent *rpoB* mutations were Ser450Leu (15.2%), Asp435Val (1.8%) and His445Tyr (1.3%) (Table S1).

**Table 1:** *Mycobacterium tuberculosis* isolates analysed (n = 32,669).

Characteristic	–	N	%
Lineage	1	3154	9.7
	2	8257	25.3
	3	3745	11.5
	4	16,684	51.1
	Other	829	2.5
Genotypic status	Sensitive	19,587	60.0
	Rifampicin resistant	7974	24.4
	Isoniazid resistant	9546	29.2
	MDR-TB	5385	16.5
	Pre-XDR-TB	2085	6.4
	XDR-TB	16	0.1
	Other drug resistance	2558	7.8

MDR-TB = multi-drug resistant; XDR-TB = extensively drug resistant.

To characterise putative novel resistance mutations, we considered samples that had a compensatory mutation, but no known resistance mutation. A manually curated list of established compensatory mutations (n = 33) (Table S2) covered *ahpC* (n = 18; e.g., -47_-46ins, -48G>A, -51G>A, -52C > A, -52C>T, -81C>T), *rpoC* (n = 13; e.g., Asn698Ser, Asp485Asn, Ile491Thr, Ile491Val, Leu516Pro, Trp484Gly, Val483Ala, Val483Gly), and *rpoA* (n = 2; e.g., Thr187Ala) loci. The number of occurrences of individual compensatory mutations within the 32 k isolates varied for *rpoC*/*A* (RIF, range: 5 – 427 isolates) and *ahpC* (INH, range: 3–97 isolates) genes. No isolate had more than one compensatory mutation for RIF or INH, and across MDR-TB.

### Putative novel resistance mutations

Using the *rpoA* and *rpoC* compensatory mutations, there were no RIF resistant isolates without a known *rpoB* resistance mutation (Figure S1). For INH, there were 561 samples with a compensatory mutation, of which 188 (33.5%) had no known *katG* resistance mutation (Figure S1). Within the 188 samples we looked for mutations in *katG* that could potentially explain the emergence of the compensatory mutation. In total, 782 unique non-synonymous mutations were found in the *katG* gene. Only 31 (4.0%) of these *katG* mutations occurred in at least three isolates, and had > 50% of isolates with a resistant DST and genotypic resistance to at least one other drug. These 31 high-quality *katG* mutations were present in 171 isolates, including 64 and 107 with and without compensatory mutations, respectively (Table 2; Figure S1). Of the 188 isolates that had a compensatory mutation, 124 (66.0%) did not have any of the 31 highly quality *katG* mutations, but 86 (/124; 69.3%) were found to have rare *katG* mutations that did not pass the minimum frequency cut-off (> = 3) used to define putative resistance mutations (Table S3). These rare *katG* mutations could also potentially explain the acquisition of a compensatory mutation but were not analysed further.

**Table 2:** List of 31 high-quality potential resistance mutations for isoniazid in the *katG* gene (171 samples).

Change	Freq	Proportion Co-occurring with a resistance mutation	Proportion Co-occurring with a compensatory mutation	Distance from heme-binding site	Predicted stability change (ΔΔG)
Trp191Gly	25	0.920	0.160	27.912	− 3.366
Ala109Thr	13	1	0.154	12.219	− 1.546
Tyr98Cys	11	0	0.091	14.835	− 2.197
Asp142Gly	9	0.222	0.222	16.149	− 1.321
Gln439His	8	1	1	25.545	− 0.839
Tyr413Cys	8	0.750	0.375	16.805	− 1.399
Gly169Ser	7	1	0.286	12.856	− 1.655
Gly299Ser	7	0.429	0.286	20.045	− 1.463
Pro232Ser	7	0.714	0.429	8.781	− 1.038
Thr677Pro	7	0.714	0.286	49.969	− 0.712
Asp189Gly	6	0.500	0.333	25.272	− 0.757
Asp419Tyr	6	0.500	0.167	21.015	− 0.688
Arg484His	5	0.200	0.200	29.488	− 2.107
Asn655Asp	5	1	0.600	55.642	− 1.589
Phe183Leu	5	0.800	0.800	21.224	− 0.833
Trp161Cys	5	0.600	1	21.583	− 2.140
Ala312Glu	4	1	1	16.287	− 1.663
Arg78Pro	4	0.500	0.500	25.94	− 0.100
Asp189Asn	4	0.500	0.250	25.272	− 0.899
Asp675Tyr	4	0.750	0.250	49.174	0.082
Glu233Gly	4	0.750	0.250	12.377	− 1.163
Gly124Ser	4	1	1	21.677	− 1.038
Ala122Asp	3	0.333	0.333	16.621	− 1.207
Arg385Pro	3	0.667	0.667	16.767	− 0.936
Gln88Pro	3	1	0.333	22.655	0.038
Gly182Arg	3	0.333	1	21.573	− 0.851
Leu132Arg	3	1	0.333	16.532	− 1.618
Met257Val	3	0.667	0.333	15.827	− 1.681
Thr271Ile	3	0	0.333	9.640	− 1.259
Thr326Pro	3	0.667	0.667	14.505	− 0.341
Trp90Arg	3	0.667	0.667	22.822	− 2.682

### Resistance and co-occurrence with other resistance mutations

**Figure 1:**
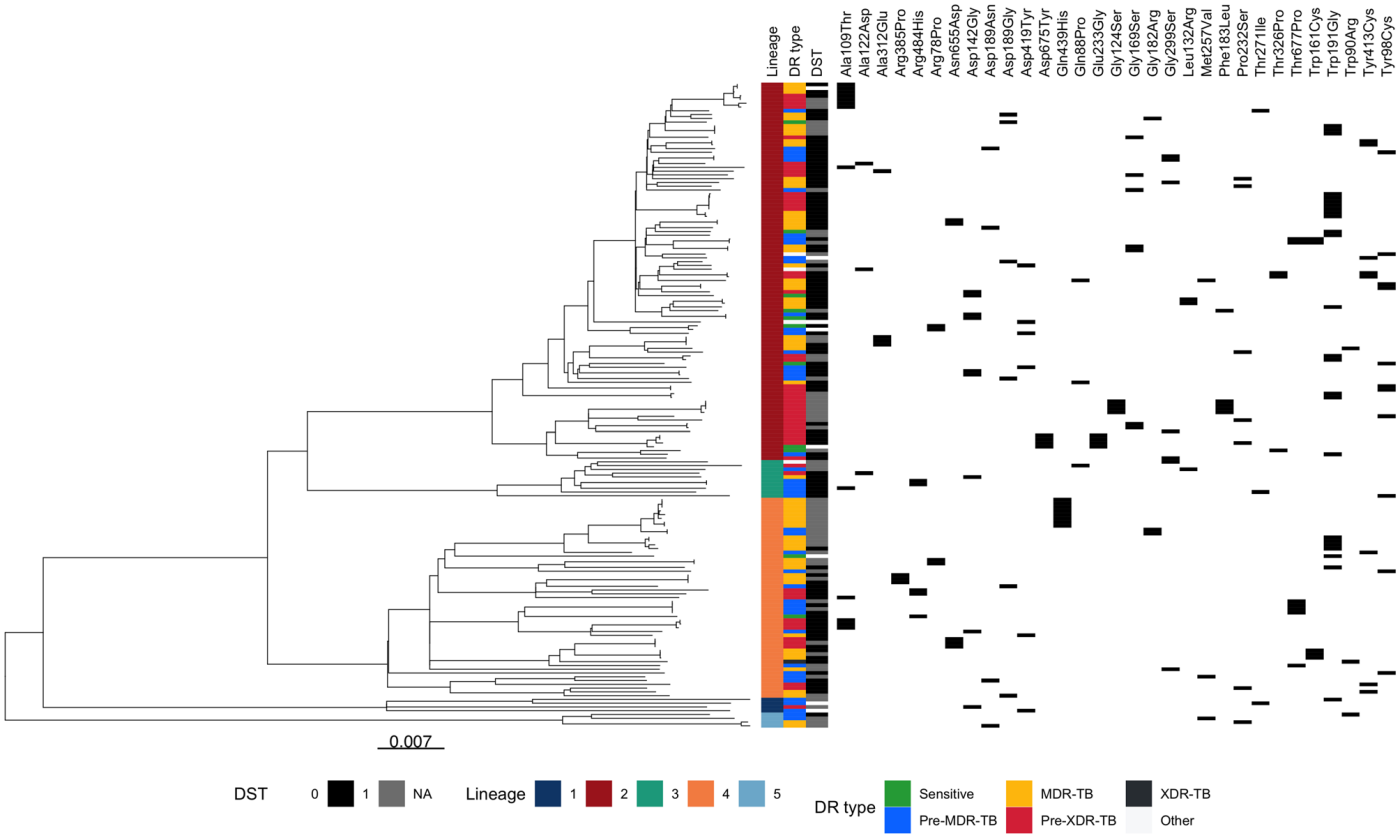
Phylogenetic tree of isolates (n = 171) with 31 putative novel *katG* gene mutations for Isoniazid resistance, with lineage, drug resistance (DR) status, and phenotypic drug susceptibility test (DST) data.

**Figure 2:**
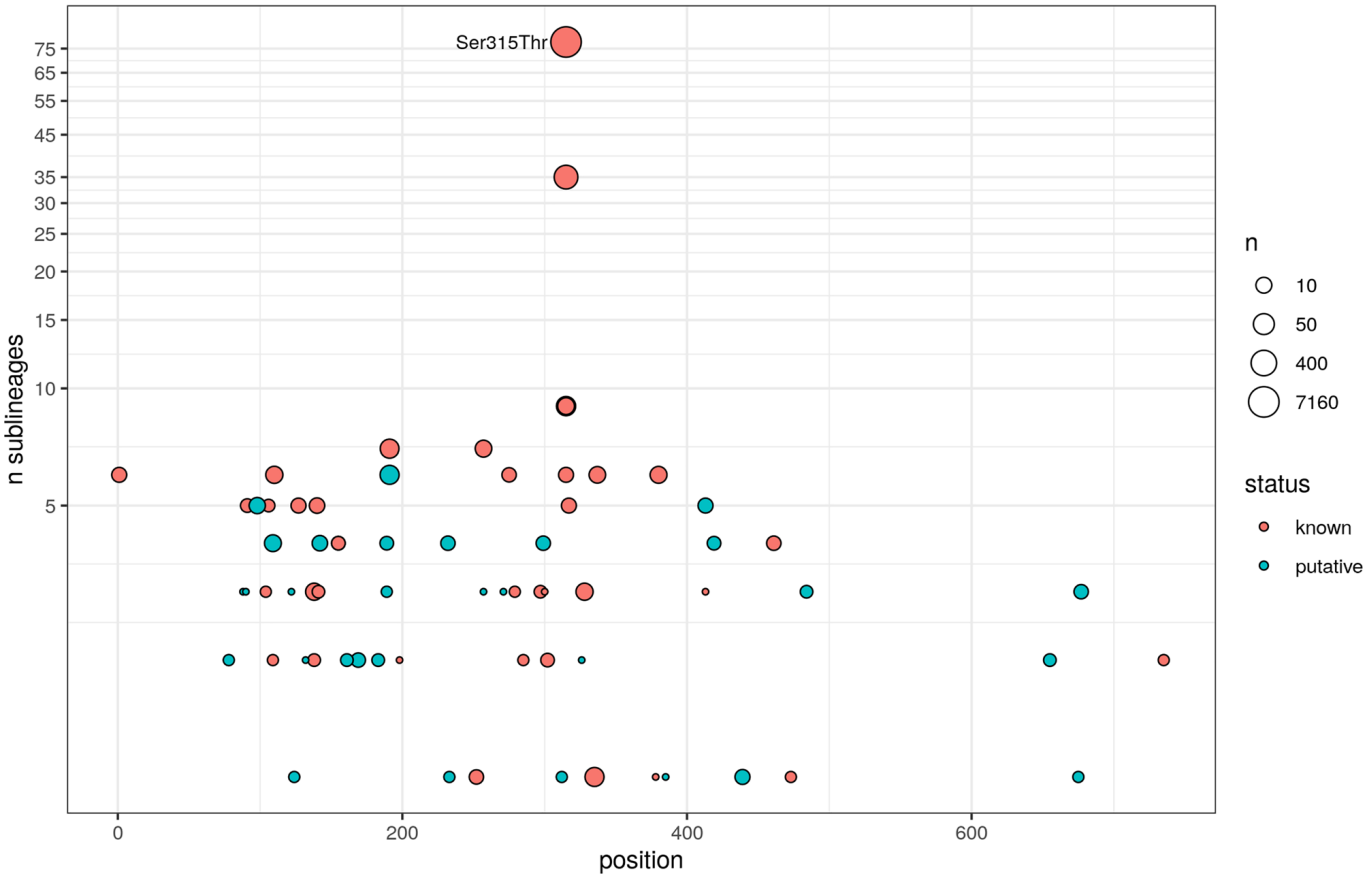
Homoplasy among 40 known *katG* and 31 putative resistance *katG* mutations. The common *katG* Ser315Thr mutation is highlighted. Mutations occurring in < 3 isolates and non-protein coding regions are omitted.

The 31 putative INH-*katG* resistance mutations occurred in multiple lineages (L1-L5) with many showing evidence of convergent evolution (Fig. 1). These putative mutations occur in similar numbers of sub-lineages and at similar *katG* gene positions compared to known resistance mutations, indicating that they show comparable phylogenetic and gene location characteristics (Fig. 2). Due to the multi-drug regimens used for TB treatment, resistance often develops to multiple drugs in a stepwise manner^13^. The co-occurrence of the 31 *katG* mutations with other resistance mutations was analysed to characterise the isolate profiles in which putative resistance mutations occur. The 31 *katG* mutations were most frequently found in isolates characterised as MDR-TB (35.1%), but also common in pre-MDR-TB (26.3%) and pre-XDR-TB (29.2%) samples. Interestingly, around half (83/171; 48.5%) of isolates with any of the 31 *katG* mutations had co-occurrence with others linked to INH, with the *fabG1* -15C>T promoter mutation being the most frequent (60/171; 35.1%) (Table S4). This observation is in stark contrast to the *katG* Ser315Thr mutation, the most prevalent resistance mutation in INH resistant isolates and known to confer a high level of resistance, which only co-occurs with other INH resistance mutations in 16.1% of isolates. Of the 171 isolates with a putative resistance mutation (Figure S1), 107 (62.6%) had an available DST result for INH, with 99 reporting a resistant phenotype leading to a highly significant association between the putative drug resistance mutations and DST phenotype (Chi-squared *P* < 1.4 × 10^–18^). Of those with a resistant DST (n = 99), 53 (53.5%) had no other known mutations that could explain resistance.

Isolates with mutations conferring a high level of drug resistance tend to have low numbers of co-occurring resistance mutations linked to that resistance. As a proxy for measuring resistance level, we calculated the proportion of known and putative resistance mutation samples with co-occurring non-*katG* (*fabG1*, *inhA*, *kasA*) resistance mutations. Mutations at the *katG* 315 codon position, which are known to confer high resistance^14^, had a relatively low proportion of isolates with co-occurring non-*katG* resistance mutations; four out of the five known codon 315 mutations have < 20% of isolates with co-occurring resistance mutations. There was no major difference in the number of co-occurring non-*katG* mutations between the putative (n = 31) and known resistance *katG* substitutions (n = 40; all > 2 isolates; Table S5) (mean resistance co-occurrence proportion: known 0.274 vs. putative mutations 0.413; T-test *P* = 0.15).

### Mutation fitness

Compensatory mutations are linked to mutations with high fitness costs (e.g., *katG* loss of function (LOF)). To estimate the fitness impact of the putative resistance mutations, the frequency of co-occurrence with a compensatory mutation was calculated. As a proof of principle, this relationship was tested by comparing the frequency of compensatory mutations in samples containing LOF mutations against those that have SNP-based resistance mutations. Having a LOF mutation is associated with an increased risk of having a compensatory mutation (odds ratio = 13.86, Chi-squared *P* < 0.0001). In general, rarer mutations were observed to co-occur more frequently with compensatory mutations (Fig. 2). The proxy fitness cost was discretised into ‘low,’ ‘medium’ and ‘high’ categories based on tertiles (see Methods). The *katG* Ser315Thr is known to confer a low fitness cost^15^, and was classified into the ‘low’ category, with only ~ 3% of samples containing the mutation co-occurring with a compensatory mutation. In fact, mutations at the codon 315 position appear to have low fitness cost (Fig. 2), where four out of the five known codon 315 resistance mutations were classified into the ‘low’ category.

**Figure 3:**
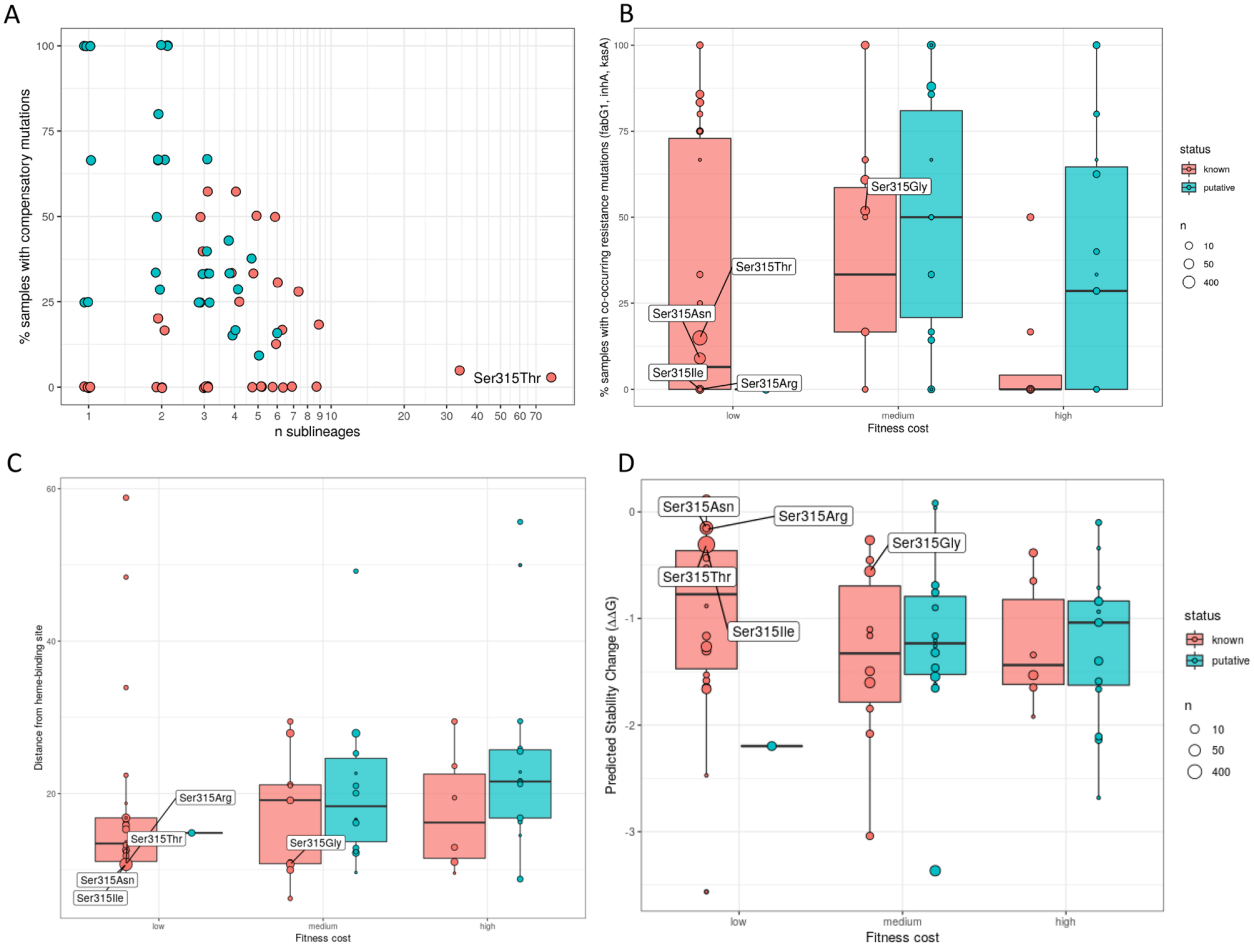
Comparison of mutation characteristics between putative and known resistance mutations. A) For each resistance mutation, the percentage of samples with a co-occurring compensatory mutation is plotted against the total number of sub-lineages it occurs in. B) Boxplot showing the percentage co-occurrence with other resistance mutations grouped by the discretised fitness categories (see Methods). Bottom boxplots show C) distance from INH heme binding site, and D) stability change distributions, grouped by the fitness categories.

Overall, compensatory mutations seem to occur in a higher proportion in isolates with the putative *katG* resistance mutations (0.388; 64/165) compared to Ser315Thr (0.031; 185/6010) (Chi-squared *P* < 10^–16^), suggesting that on average they incur a greater fitness cost compared to this high frequency global mutation. Similarly, comparing to the 40 known resistance *katG* mutations from above (Table S5), there was a higher proportion of isolates with a compensatory mutation in those with the putative mutations (proportion of isolates with compensation mutation: known 0.026 vs. putative 0.389; Chi-squared *P* = 6 × 10^–5^). This difference remained statistically significant even when excluding the codon 315 positions (Chi-squared *P* = 4 × 10^–4^). Further, there appears to be little association between known non*-katG* resistance mutation co-occurrence (resistance level) and proxy fitness cost in both the 40 known and the 31 putative *katG* resistance mutations (Linear model *P* = 0.073) (Table S5). Also, across each of the three fitness cost categories (high, medium, and low), there were no strong differences in resistance mutation co-occurrence (resistance level) between isolates with the known and putative *katG* resistance mutations (minimum *P* = 0.144; Fig. 3). No strong differences in the co-variation between resistance level and fitness cost across known and putative mutations supports the veracity of our putative resistance variants. Interestingly, it has previously been observed that RIF-associated compensatory mutations in *rpoC* co-occur most frequently with *rpoB* Ser450Leu, which is the most common RIF resistance mutation and is thought to have a low fitness impact. This observation was also confirmed in our analysis, where 24.5% of the 4970 samples with the *rpoB* Ser450Leu mutation had a compensatory mutation. This was followed by Gln432Lys (19.2%), Val170Phe (16.4%), Gln432Leu (14.3%) and Pro454His (14.3%) (Table S6). Only Gln432Pro had a higher percentage co-occurrence, with 41.9% of 31 samples with this mutation also having a compensatory mutation.

### Protein structure modelling

To explore the functional effects of the 31 putative resistance mutations, in silico predictions of their effects on the *katG* target protein were assessed (Fig. 3, Table 2). The estimable distances from the *katG* heme-binding site, thought to be close to the active INH binding site and crucial to enzymatic activity^16^, did not differ significantly between known and putative substitution resistance mutations (Table S5) (mean distance: known 26.626 A^o^ vs. putative 22.058 A^o^; Wilcoxon *P* = 0.06). There was no significant difference in protein stability change between the known resistance and putative *katG* mutations (mean $\Delta \Delta$ G: known − 1.078 vs. putative − 1.257; Wilcoxon *P* = 0.24).

## Discussion

Our goal was to identify putative novel mutations underlying resistance to RIF and INH by finding isolates with established compensatory mutations. No novel *rpoB* gene mutations potentially linked to resistance to RIF were found, but this may be expected since there are limited ways in which the precise machinery of RNA polymerase can change without a loss of function. In contrast, many changes in the KatG protein can leave the bacteria largely unaffected. Our methodology flagged 31 mutations in *katG* that were analysed further. Evidence from available phenotypic DST data strongly suggests that the 31 *katG* mutations identified confer resistance. These mutations occur in multiple sub-lineages and independently in the phylogeny, a pattern of convergent evolution that is well established in known *katG* resistance mutations. Due to the relative rare occurrence of these mutations, they are either not present in the WHO catalogue or they have been designated as uncertain significance. However two of the mutations (Gly169Ser^17^ and Asp142Gly^17,18^) were previously designated as likely to explain resistance in clinical isolates. Whilst our analysis focused on 31 high quality and frequent putative mutations in *katG*, less common mutations identified in one or two isolates may be of interest, including for functional evaluation and surveillance applications.

No significant differences were found in the proportion of isolates with (non-*katG*) known resistance mutation co-occurrence (our proxy for resistance level) between the filtered known (n = 40) and putative (n = 31) resistance mutations. In showing a similar pattern of resistance mutation co-occurrence we infer that the putative resistance mutations confer on average a similar level of resistance to known mutations, and this further supports their causal role with resistance. There was, however, a difference in the fitness cost between the known and putative resistance mutations, measured using compensatory mutation co-occurrence, with the latter appearing to have on average a higher cost. This observation is in agreement with previous studies, which report higher co-occurrence of *ahpC* promoter mutations with non-315 *katG* mutations compared to codon 315 mutations^19^. Whilst known resistance mutations are likely to converge on the most stable protein configurations and hence proliferate, the putative mutations are rarer and less likely to have been previously associated with drug resistance. The Interpretation of fitness cost and its relationship to compensatory mutations is less clear for *rpoB*/*C*/*A* (RIF) compared to *katG*/*ahpC* (INH). For example, *rpoB* Ser450Leu is thought to incur a minor fitness cost, yet compensatory mutations are found most frequently with this mutation. Conversely, *rpoB* Asp435Gly is described as having a 'severe' fitness cost^20^, yet in our data none of the 90 samples with this mutation have compensatory *rpoC* mutations. Interestingly, three of these five mutations occur at position Gln432, indicating that mutations at this codon are heavily associated with having a compensatory mutation. There was no relationship between resistance level (using co-occurrence with other resistance mutations as a proxy) and fitness cost in either the 40 known or 31 putative filtered resistance mutations. Again, this similar pattern of variation indicates the veracity of the putative resistance mutations. Further, the functional impact of the 31 putative *katG* mutations is supported by in silico protein modelling, with distances to the functionally important *katG* heme active binding site similar to those of known variants, indicating that they are likely confer a similar pattern of resistance. In contrast to the differences between known and putative mutations in their percentages of isolates with compensatory mutations, surprisingly, there was no difference in the *in-silico* $\Delta \Delta$ G measure predictions. However, the $\Delta \Delta$ G measure is an indicator of protein stability, and therefore only an indirect indication of fitness cost.

There is the opportunity to apply a similar approach to other forms of Mtb drug resistance with a compensatory-resistance dynamic. This is especially true for non-essential targets that can exhibit multiple resistance mutations without a loss of function, similar to *katG*. For example, compensatory mutations for streptomycin are purported to restore translational accuracy of the ribosome, the target of the anti-TB drug^21^. Similarly, compensatory mutations have been found to act upon structures intolerant to change, including DNA gyrase subunit A (*gyrA* gene) for fluoroquinolones, and 16S rRNA of the 30S ribosome subunit (*rrs* gene) for aminoglycosides (e.g., capreomycin^20^). Ultimately, through identifying the full repertoire of resistance and compensatory mutations for anti-TB drugs, there will be improvements in clinical management and surveillance decision making using whole genome and amplicon sequencing data.

## Conclusions

We have presented an approach to identify potential resistance mutations to monitor the development of resistance mechanisms to important first-line isoniazid and rifampicin anti-TB drugs, and therefore MDR-TB. The list of putative resistance mutations can inform functional studies of resistance, and after validation, be incorporated into genotypic drug resistance prediction, thereby informing clinical and infection control activities.

## Material and methods

### Input data and processing

The main input data consists of a database of 32 k isolates with DST and sequence data has been described previously^22^. Sequences were aligned to the H37Rv reference genome^23^ (AL123456) with BWA mem (v0.7.17) software^24^. Joint SNP and indel calling was carried out in gatk GenotypeGVCFs (v4.1.3.0) software^25^. Monomorphic SNP/indel variants and those in non-unique regions of the genome (e.g., *ppe* genes) were excluded. Multi-FASTA alignments were created from the filtered variant and H37Rv reference fasta files using bedtools makewindows (v2.28.0)^26^ and custom python scripts. Phylogenetic trees were constructed using IQ-TREE (v1.6.12) software, applying a general time reversible model with rate heterogeneity set to a discrete gamma model and an ascertainment bias correction (parameters − m GTR + G + ASC), with 1000 bootstrap samples^27^. Drug resistance types and lineages were predicted in-silico with TB-Profiler (v4.3.0) software^6,28^. TB-Profiler software was also used to identify all known drug resistance, compensatory and putative novel resistance mutations. Resistance patterns of samples were determined using phenotypic DSTs (available for 54% of samples) and predictions from TB-Profiler software (available for all samples). These resistance patterns were used to filter mutations (as described below). Known resistance mutations were defined based on the manually curated TBDB database (version commit: 4,738,132) which contains all WHO-endorsed mutations and additional ones reported in the literature.

### Finding putative resistance markers using compensatory mutations

To improve the power of the analysis, novel compensatory mutations in *ahpC* were first characterised, as they are less well established than those in *rpoC/A*. From the sequence database (n = 32 k), all non-synonymous mutations present in at least three samples were found in *ahpC*. Although compensatory mutations do not cause resistance, they are strongly associated. Therefore, all mutations were filtered with requirements that > 50% samples were predicted resistant to INH by TB-Profiler and > 50% of samples were INH DST resistant. As there were many potential *ahpC* mutations, further filtering criteria were applied to these. Specifically, mutations were retained if they were associated with a loss of function in *katG* mutations, occurred in the same position as known *ahpC* mutations, and if they appeared in multiple lineages (convergent evolution). Only one of these criteria needed to be met to be considered a potential compensatory *ahpC* mutation. The full list of compensatory mutations consisted of 31 mutations (Table S1). A proxy for fitness cost was based on tertiles of the percentage of samples with compensatory mutations (low: <  = 17%, medium: > 17%  and <  = 40%, high: > 40%  and <  = 100%). To find putative resistance mutations, all non-synonymous mutations in the relevant resistance genes were extracted from the TB-Profiler database (*katG* for INH, *rpoB* for RIF). Some variants known not to be associated with INH resistance^29^ (e.g., *katG*-Arg463Leu) were excluded.

For each drug, mutations were found in samples where a compensatory mutation was present and known resistance mutations were absent in the relevant genes, but a non-resistance-associated mutation was present in the relevant target genes. These mutations were then filtered to exclude known drug-resistance-associated variants, and subjected to the same criteria as the putative compensatory mutations i.e., present in three or more samples, < 50% samples were predicted sensitive by TB-Profiler and < 50% of samples were DST sensitive. Mutations occurring in promoter regions of *rpoB* were excluded as candidate potential resistance mutations, as there is no known mechanism of resistance that could result from increased expression of the RNA polymerase beta subunit. Using this list of potential resistance mutations, all TB-Profiler database isolates (n = 32 k) were then searched for their presence, regardless of compensatory mutation status. It should be noted that therefore not all samples with potential resistance mutations necessarily have compensatory mutations, and vice versa.

### Protein structural modelling

The open source software ChimeraX^30^ was used to model distances from the INH heme binding site. Effects of mutations on protein stability were predicted using *in-silico* changes in Gibbs free energy ($\Delta \Delta$ G) by mCSM^31^ software.

## Supplementary Information

Supplementary Information.

## Data Availability

All genomic data is available on the short read archive (https://www.ebi.ac.uk/ena/browser/). Code and accessions used in the study can be found at https://github.com/GaryNapier/comp_mut. Analysis scripts are available at https://github.com/AntonS-bio.
