# Supplementary material for: Large-scale genomic analysis of Mycobacterium tuberculosis reveals extent of target and compensatory mutations linked to multi-drug resistant tuberculosis
Source: Sci Rep. 2023 Jan 12;13:623. doi: 10.1038/s41598-023-27516-4 (PMC9837068; doi:10.1038/s41598-023-27516-4)

## SUPPLEMENTARY TABLES

**Table S1**

**Common established resistance mutations for INH and RIF*. Mutations occurring in <5 isolates in the 32k dataset are omitted.**

| Drug | Gene | Mutation [frequency] |
| --- | --- | --- |
| isoniazid | *katG* | Ser315Thr [7165], Ser315Asn [167], Ser315Gly [27], Ile335Val [25], Trp191Arg [25], Ala110Val [14], Asn138His [14], Trp328Leu [14], Ser315Ile [13], Thr380Ile [13], 371del [12], Met257Ile [12], Tyr337Cys [12], 1284del [9], Ser140Asn [9], Gln127Pro [8], Ile317Val [8], Ser315Arg [8], Val1Ala [8], Gln461Pro [7], Phe252Leu [7], Thr275Ala [7], Tyr155Cys [7], Ser302Arg [6], Trp91Arg [6], Tyr155Ser [6], 18dup [5], Ala106Val [5], Asn138Ser [5], Gly297Val [5], Leu141Phe [5] |
|  | *fabG1* | -15C>T [1989], -17G>T [173], -8T>A [159], -8T>C [154], -8T>G [11] |
|  | *inhA* | -154G>A [332], Ser94Ala [234], Ile194Thr [191], Ile21Thr [103], Ile21Val [94] |
|  | *ahpC* | Asp73His [50], Glu76Lys [14] |
| rifampicin | *rpoB* | Ser450Leu [4970], Asp435Val [592], His445Tyr [410], His445Asp [293], Asp435Tyr [277], Leu452Pro [251], Glu761Asp [227], Leu430Pro [140], Ser450Trp [130], Ile491Phe [126], His445Leu [101], His445Arg [98], Asp435Gly [95], His445Asn [95], Val170Phe [73], Ser450Phe [46], Asp435Phe [41], Ser441Leu [39], His445Cys [36], Ala286Val [33], Gln432Pro [31], Ile480Val [29], Gln432Lys [26], Met434Ile [25], 1297_1299dup [24], Gln432Leu [21], Ser441Gln [21], Thr400Ala [21], His445Gln [20], Leu430Arg [18], Asp435Ala [14], 1296_1304del [12], Asn437Asp [12], Gln432Glu [10], 1329_1331dup [9], Gln429His [9], His445Gly [9], Ser431Gly [9], Ser450Gln [9], His445Ser [8], Ser428Arg [8], 1312_1314del [7], Asp435Glu [7], Phe424Val [7], Pro454His [7], Pro454Leu [7], Ser493Leu [7], Ala451Val [6], Glu460Gly [6], Met434Val [6], Phe424Leu [6], 1287_1295del [5], 1300_1305del [5], Gln429Leu [5], Ser428Gly [5] |

* from TB-Profiler

**Table S2**

**Mutations in compensatory genes and their frequencies in 32k samples. All mutations occur in >1 lineage.**

| Drug | Compensatory locus | Mutation [frequency] |
| --- | --- | --- |
| isoniazid | *ahpC* | -47_-46ins [49*], -48G>A [92], -51G>A [44*], -52C>A [49*], -52C>T [97], -54C>T [33], -57C>T [26], -72C>T [37*], -74G>A [9*], -75T>G [3*], -76T>A [25*], -76T>G [5*], -77del [9*], -77T>A [6*], -77T>G [10*], -81C>T [57], -88_-87ins [4*], -90G>A [8*] |
| rifampicin | *rpoA* | Thr187Ala [59], Thr187Pro [6] |
|  | *rpoC* | Asn698His [10], Asn698Lys [17], Asn698Ser [125], Asp485Asn [48], Asp485His [5], Ile491Thr [107], Ile491Val [161], Leu516Pro [66], Phe452Leu [25], Pro434Arg [11], Trp484Gly [53], Val483Ala [135], Val483Gly [427] |

[frequencies]; ins = insertion, del = deletion; * novel markers with strong evidence for compensatory effects through convergent evolution, co-occurrence with loss of function mutations in *katG* as well as association with INH resistant isolates.

**Table S3**

**Less frequent mutations (n=84) in *katG* (<3 isolates) in 86 isolates with no known isoniazid resistance mutations, with compensatory mutations but no potential resistance mutation****

| Change | Frequency | # Co-occurring with a resistance mutation | # Co-occurring with a compensatory mutation | Distance from heme-binding site | Predicted Stability Change (ΔΔG) |
| --- | --- | --- | --- | --- | --- |
| Leu43Arg | 2 | 0 | 2 | 38.063 | -1.520 |
| Leu48Arg | 1 | 0 | 1 | 35.410 | -1.260 |
| Leu76Pro | 1 | 0 | 1 | 24.637 | -1.373 |
| Thr86Pro | 2 | 0 | 2 | 22.091 | -0.490 |
| Ala93Thr | 2 | 0 | 2 | 17.033 | -1.007 |
| His97Pro | 1 | 0 | 1 | 15.693 | -0.074 |
| Ile103Val | 2 | 0 | 1 | 7.7860 | -1.467 |
| Gly111Asp | 1 | 0 | 1 | 11.404 | -1.512 |
| Ala122Val | 1 | 0 | 1 | 18.105 | -0.689 |
| Gly124Ala | 1 | 0 | 1 | 21.029 | -0.723 |
| Phe129Ser | 1 | 0 | 1 | 20.644 | -2.619 |
| Trp135* | 1 | 0 | 1 | - | - |
| Pro136Leu | 2 | 2 | 2 | 11.891 | -0.267 |
| Leu141Val | 1 | 1 | 1 | 13.825 | -1.872 |
| Asp142Asn | 1 | 0 | 1 | 16.634 | -1.387 |
| Lys143Asn | 1 | 0 | 1 | 16.183 | -1.704 |
| Arg145Ser | 1 | 1 | 1 | 18.166 | -2.084 |
| Gly156Asp | 1 | 0 | 1 | 29.539 | -1.595 |
| Ala162Val | 3 | 1 | 1 | 19.634 | -0.804 |
| Asp163Asn | 1 | 0 | 1 | 19.888 | -0.609 |
| Asp163Ala | 1 | 0 | 1 | 19.888 | -0.788 |
| Ile165Thr | 1 | 0 | 1 | 15.343 | -2.971 |
| Phe167Ser | 1 | 0 | 1 | 17.090 | -3.291 |
| Leu173Arg | 2 | 0 | 2 | 15.564 | -1.872 |
| Gly184Asp | 1 | 0 | 1 | 23.169 | -2.176 |
| Gly186Ser | 2 | 0 | 2 | 25.211 | -1.465 |
| Gly186Asp | 1 | 0 | 1 | 25.211 | -1.924 |
| Met225Ile | 1 | 1 | 1 | 16.258 | -0.154 |
| Thr251Lys | 1 | 1 | 1 | 13.673 | -0.467 |
| Arg253Trp | 2 | 2 | 2 | 17.457 | -0.390 |
| Thr262Pro | 2 | 0 | 2 | 11.530 | -0.237 |
| Ala264Val | 2 | 2 | 1 | 10.974 | 0.230 |
| Gly273Arg | 4 | 0 | 1 | 8.9660 | -0.981 |
| His276Gln | 1 | 0 | 1 | 12.199 | -0.662 |
| Glu289Ala | 2 | 2 | 1 | 24.324 | -0.982 |
| Gly299Asp | 1 | 0 | 1 | 19.291 | -1.975 |
| Ala312Val | 1 | 0 | 1 | 15.602 | -0.590 |
| Thr324Leu | 1 | 0 | 1 | 14.819 | -0.392 |
| Pro325Ser | 2 | 0 | 1 | 12.972 | -2.410 |
| Trp328Arg | 1 | 0 | 1 | 16.309 | -2.336 |
| Asp329Ala | 2 | 1 | 1 | 16.298 | -0.523 |
| Asp329Glu | 2 | 0 | 2 | 16.298 | -0.604 |
| Glu342Gly | 1 | 0 | 1 | 19.479 | -1.412 |
| Thr344Ser | 1 | 0 | 1 | 18.705 | -1.089 |
| Ser383* | 1 | 1 | 1 | - | - |
| Thr394Pro | 1 | 0 | 1 | 18.440 | -0.410 |
| His400Pro | 1 | 0 | 1 | 21.679 | 0.426 |
| Phe408Ser | 1 | 0 | 1 | 14.601 | -2.775 |
| Ala411Asp | 1 | 0 | 1 | 14.011 | -2.547 |
| Tyr413Ser | 1 | 0 | 1 | 16.985 | -3.198 |
| Asp419Val | 1 | 0 | 1 | 21.844 | 0.183 |
| Pro422Leu | 1 | 0 | 1 | 28.127 | -0.371 |
| Tyr426* | 1 | 0 | 1 | - | - |
| Leu458His | 1 | 0 | 0 | 55.573 | -2.819 |
| Ile462Ser | 1 | 1 | 1 | 56.503 | -3.388 |
| Ala476Glu | 1 | 0 | 1 | 43.138 | -2.446 |
| Ala478Arg | 1 | 0 | 1 | 39.741 | -0.866 |
| Ala480Gln | 2 | 0 | 2 | 37.360 | -1.471 |
| Phe483Leu | 2 | 0 | 1 | 31.498 | -1.654 |
| Lys488Glu | 2 | 0 | 2 | 27.512 | -0.682 |
| Gly490Asp | 2 | 0 | 1 | 32.196 | -0.930 |
| Gly494Ala | 2 | 0 | 2 | 37.761 | -0.959 |
| Gly495Ser | 8 | 0 | 1 | 39.032 | -1.750 |
| Gly495Cys | 2 | 0 | 2 | 39.032 | -1.578 |
| Pro501Ser | 2 | 2 | 2 | 35.629 | -2.376 |
| Leu521Pro | 1 | 0 | 1 | 50.801 | -1.520 |
| Gly560Arg | 1 | 0 | 1 | 62.465 | -0.240 |
| Thr568Pro | 2 | 2 | 1 | 44.809 | -0.224 |
| Pro569Leu | 1 | 0 | 1 | 44.191 | -0.400 |
| Asp612Gly | 1 | 0 | 1 | 32.396 | -0.696 |
| Ala621Asp | 1 | 0 | 1 | 42.314 | -2.436 |
| Thr625Lys | 2 | 0 | 2 | 46.002 | 0.112 |
| Leu627Pro | 1 | 0 | 1 | 45.368 | -0.904 |
| Gly630Arg | 1 | 0 | 1 | 49.604 | -0.760 |
| Gly644Asp | 2 | 1 | 1 | 52.172 | -1.423 |
| Asp663Tyr | 2 | 1 | 1 | 59.875 | -0.018 |
| Gln679Tyr | 1 | 0 | 1 | 57.006 | -0.175 |
| Ser700Phe | 1 | 1 | 1 | 48.170 | -0.948 |
| Arg705Trp | 1 | 0 | 1 | 50.045 | -1.636 |
| Val708Asp | 1 | 0 | 1 | 52.230 | -2.899 |
| Tyr711Asp | 2 | 0 | 2 | 54.722 | -3.780 |
| Asp723Asn | 1 | 0 | 1 | 48.405 | -1.170 |
| Asp735Tyr | 1 | 0 | 1 | 34.481 | -0.253 |
| Arg736Lys | 1 | 0 | 1 | 35.676 | -1.420 |

* stop codon; ** see Methods for definition

**Table S4**

**Known resistance and other mutations co-occurring in isolates with the 31 putative drug resistance mutations**

| Putative resistance mutations (*katG*) | Known resistance mutations | Other mutations | n |
| --- | --- | --- | --- |
| Ala109Thr | fabG1-15C>T | - | 7 |
| Ala109Thr | fabG1-17G>T | - | 1 |
| Ala109Thr | fabG1-8T>A | katG-Val697Ala | 3 |
| Ala109Thr | inhA-Ile194Thr; fabG1-15C>T | - | 2 |
| Ala122Asp | inhA-154G>A | - | 1 |
| Ala122Asp | - | - | 2 |
| Ala312Glu | - | kasA-Val142Ile | 1 |
| Ala312Glu | - | - | 3 |
| Arg385Pro | - | - | 3 |
| Arg484His | - | kasA-Gly269Ser | 1 |
| Arg484His | - | - | 4 |
| Arg78Pro | - | - | 4 |
| Asn655Asp | fabG1-15C>T | - | 2 |
| Asn655Asp | - | - | 3 |
| Asp142Gly | katG-Ser140Asn | - | 1 |
| Asp142Gly | - | - | 8 |
| Asp189Asn | fabG1-15C>T | - | 2 |
| Asp189Asn | - | - | 2 |
| Asp189Gly | fabG1-15C>T | - | 1 |
| Asp189Gly | - | katG-Ser446Asn | 1 |
| Asp189Gly | - | - | 4 |
| Asp419Tyr | fabG1-15C>T | - | 1 |
| Asp419Tyr | fabG1-8T>C | - | 1 |
| Asp419Tyr | - | kasA-Gly312Ser | 1 |
| Asp419Tyr | - | - | 3 |
| Asp675Tyr; Glu233Gly | fabG1-15C>T | katG-Thr380Ala | 2 |
| Asp675Tyr; Glu233Gly | - | - | 1 |
| Asp675Tyr; Pro232Ser; Glu233Gly | - | - | 1 |
| Gln439His | fabG1-15C>T | - | 8 |
| Gln88Pro | fabG1-15C>T | katG-Lys600Gln | 1 |
| Gln88Pro | inhA-154G>A | - | 1 |
| Gln88Pro; Met257Val | - | kasA-His253Tyr | 1 |
| Gly169Ser | fabG1-15C>T | - | 6 |
| Gly169Ser | - | - | 1 |
| Gly182Arg | - | - | 3 |
| Gly299Ser | fabG1-15C>T | - | 1 |
| Gly299Ser | - | katG-Gln525Leu | 1 |
| Gly299Ser | - | - | 5 |
| Leu132Arg | fabG1-15C>T | katG-Val246Gly | 1 |
| Leu132Arg | fabG1-15C>T | - | 2 |
| Met257Val | katG-Gln461Pro | - | 1 |
| Met257Val | - | katG-Tyr28Leu | 1 |
| Phe183Leu | - | - | 1 |
| Phe183Leu; Gly124Ser | fabG1-15C>T | - | 4 |
| Pro232Ser | fabG1-15C>T | - | 2 |
| Pro232Ser | - | katG-Asp419Gly | 1 |
| Pro232Ser | - | katG-Gln295Glu | 1 |
| Pro232Ser | - | - | 2 |
| Thr271Ile | - | - | 3 |
| Thr326Pro | - | - | 1 |
| Thr326Pro; Tyr413Cys | fabG1-8T>C | - | 2 |
| Thr677Pro | inhA-154G>A | - | 1 |
| Thr677Pro | katG-Ser315Thr | - | 4 |
| Thr677Pro; Trp161Cys | - | - | 2 |
| Trp161Cys | - | - | 3 |
| Trp191Gly | fabG1-15C>T | katG-Val320Ala | 1 |
| Trp191Gly | fabG1-15C>T | - | 17 |
| Trp191Gly | fabG1-8T>C | - | 2 |
| Trp191Gly | inhA-154G>A | katG-Thr625Ala | 2 |
| Trp191Gly | - | - | 3 |
| Trp90Arg | - | - | 3 |
| Tyr413Cys | fabG1-15C>T | - | 2 |
| Tyr413Cys | inhA-Ser94Ala; fabG1-15C>T | - | 1 |
| Tyr413Cys | - | katG-Trp438Gly | 1 |
| Tyr413Cys | - | - | 2 |
| Tyr98Cys | - | katG-Leu378Met | 1 |
| Tyr98Cys | - | - | 10 |

**Table S5**

**Proportions of co-occurring known resistance and compensatory mutations, distance from heme binding sit and predicted stability change for the known *katG* resistance mutations. Co-occurring known resistance mutations are in *fabG1*, *inhA*, and *kasA* genes.**

| Change | Freq | Proportion  co-occurring with a resistance mutation | Proportion  co-occurring with a compensatory mutation | Distance from heme-binding site | Predicted Stability Change (ΔΔG) |
| --- | --- | --- | --- | --- | --- |
| Ser315Thr | 7163 | 0.16 | 0.02 | -0.306 | 11.693 |
| Ser315Asn | 167 | 0.10 | 0.05 | -0.150 | 11.693 |
| Ser315Gly | 27 | 0.52 | 0.11 | -0.558 | 11.693 |
| Trp191Arg | 25 | 0.56 | 0.28 | -1.602 | 27.925 |
| Ile335Val | 25 | 0.04 | 0.00 | -1.263 | 16.565 |
| Ala110Val | 14 | 0.86 | 0.00 | -0.619 | 11.992 |
| Asn138His | 14 | 0.00 | 0.57 | -1.532 | 11.675 |
| Trp328Leu | 14 | 0.00 | 0.00 | -1.662 | 16.309 |
| Thr380Ile | 13 | 1.00 | 0.31 | -0.265 | 9.721 |
| Ser315Ile | 13 | 0.00 | 0.00 | -0.340 | 11.693 |
| Met257Ile | 12 | 0.83 | 0.00 | -1.301 | 16.356 |
| Tyr337Cys | 12 | 0.17 | 0.17 | -1.494 | 20.164 |
| Ser140Asn | 9 | 0.44 | 0.44 | -0.384 | 12.600 |
| Val1Ala | 8 | 0.00 | 0.38 | - | - |
| Ile317Val | 8 | 0.00 | 0.00 | -1.272 | 12.165 |
| Gln127Pro | 8 | 0.75 | 0.00 | 0.121 | 16.793 |
| Tyr155Cys | 7 | 0.29 | 0.57 | -1.648 | 29.829 |
| Ser315Arg | 7 | 0.00 | 0.00 | -0.165 | 11.693 |
| Thr275Ala | 7 | 0.00 | 0.00 | -1.165 | 10.011 |
| Gln461Pro | 7 | 0.29 | 0.00 | -0.153 | 57.763 |
| Phe252Leu | 7 | 1.00 | 0.00 | -1.645 | 14.614 |
| Ser302Arg | 6 | 0.17 | 0.17 | -0.453 | 22.561 |
| Trp91Arg | 6 | 0.17 | 0.33 | -2.081 | 19.039 |
| Tyr155Ser | 6 | 0.67 | 0.33 | -3.040 | 29.829 |
| Leu141Phe | 5 | 0.00 | 0.00 | -1.583 | 13.825 |
| Ala106Val | 5 | 0.80 | 0.00 | -0.433 | 10.162 |
| Asn138Ser | 5 | 0.00 | 0.20 | -1.846 | 11.675 |
| Gly297Val | 5 | 0.00 | 0.40 | -0.648 | 22.251 |
| Ala109Val | 4 | 0.00 | 0.00 | -0.574 | 12.583 |
| Asp419His | 4 | 0.50 | 0.00 | -1.161 | 21.844 |
| Arg104Gln | 4 | 0.00 | 0.25 | -1.103 | 7.187 |
| Gly279Asp | 4 | 0.00 | 0.00 | -1.342 | 19.236 |
| Val473Phe | 4 | 0.00 | 0.00 | -1.529 | 47.209 |
| Gly285Asp | 4 | 0.75 | 0.00 | -0.526 | 21.774 |
| Asp735Ala | 4 | 0.25 | 0.00 | -0.663 | 34.481 |
| Tyr413His | 3 | 0.67 | 0.00 | -2.472 | 16.985 |
| Trp300Gly | 3 | 0.00 | 0.00 | -3.564 | 19.266 |
| Trp198* | 3 | 0.00 | 0.67 | - | - |
| Gly234Arg | 3 | 0.00 | 0.00 | -0.883 | 14.333 |
| Leu378Pro | 3 | 0.00 | 1.00 | -1.921 | 10.44 |
| Trp204* | 2 | 0.00 | 0.00 | - | - |
| Trp668* | 2 | 0.00 | 0.50 | - | - |
| Arg249Cys | 2 | 0.00 | 0.00 | -1.457 | 15.705 |
| Met126Ile | 2 | 1.00 | 0.00 | -0.859 | 18.132 |
| Ala139Pro | 2 | 0.00 | 0.00 | -0.599 | 11.833 |
| Glu588* | 2 | 0.00 | 1.00 | - | - |
| Trp412* | 2 | 0.00 | 1.00 | - | - |
| Ala172Val | 2 | 0.50 | 1.00 | -0.474 | 14.531 |
| Ala424Gly | 2 | 0.00 | 0.00 | -0.676 | 30.436 |
| Ala264Thr | 2 | 0.00 | 0.00 | -1.175 | 10.974 |
| Trp505* | 2 | 0.00 | 0.50 | - | - |
| Ser700Pro | 2 | 0.00 | 1.00 | -0.074 | 48.17 |
| Asn138Asp | 2 | 0.50 | 0.00 | -2.008 | 11.675 |
| Gly299Cys | 2 | 0.00 | 1.00 | -0.808 | 19.291 |
| Glu195Lys | 2 | 0.00 | 0.00 | -0.258 | 24.749 |
| Met257Thr | 2 | 0.00 | 1.00 | -2.068 | 16.356 |
| Thr85Pro | 2 | 0.00 | 0.00 | -0.315 | 22.097 |
| Ala424Val | 2 | 0.00 | 0.00 | -0.169 | 30.436 |
| Trp149* | 2 | 0.00 | 1.00 | - | - |
| Trp438* | 2 | 0.00 | 0.50 | - | - |
| Gln525Pro | 2 | 0.50 | 0.00 | 0.101 | 52.663 |
| Trp90* | 2 | 0.00 | 0.50 | - | - |
| Ala65Thr | 1 | 0.00 | 0.00 | -0.913 | 32.704 |
| Asp357His | 1 | 0.00 | 0.00 | 0.433 | 26.139 |
| Trp321* | 1 | 0.00 | 0.00 | - | - |
| Thr324Pro | 1 | 0.00 | 1.00 | -0.467 | 14.819 |
| Arg463Trp | 1 | 1.00 | 0.00 | -0.593 | 59.168 |
| Trp328Ser | 1 | 0.00 | 1.00 | -3.368 | 16.309 |
| Leu384Arg | 1 | 0.00 | 0.00 | -2.321 | 15.103 |
| Thr308Pro | 1 | 0.00 | 1.00 | -0.769 | 19.476 |
| Arg418* | 1 | 0.00 | 0.00 | - | - |
| Phe567Ser | 1 | 1.00 | 0.00 | -2.448 | 47.080 |
| Ala550Asp | 1 | 0.00 | 0.00 | -2.005 | 53.786 |
| Gln295Pro | 1 | 0.00 | 0.00 | 0.021 | 25.611 |
| Thr394Ala | 1 | 0.00 | 0.00 | -1.272 | 18.44 |
| Tyr28* | 1 | 0.00 | 0.00 | - | - |
| Gln88* | 1 | 0.00 | 0.00 | - | - |
| Thr275Pro | 1 | 0.00 | 1.00 | -0.475 | 10.011 |
| Asp311Gly | 1 | 0.00 | 1.00 | -0.970 | 18.332 |
| Ala162Thr | 1 | 1.00 | 0.00 | -1.914 | 19.634 |
| Ala379Val | 1 | 0.00 | 1.00 | -0.495 | 11.387 |
| Trp328Cys | 1 | 0.00 | 0.00 | -2.115 | 16.309 |
| Ser175* | 1 | 0.00 | 0.00 | - | - |
| Ser671* | 1 | 0.00 | 0.00 | - | - |
| Gly299Ala | 1 | 0.00 | 1.00 | -0.794 | 19.291 |
| Ser17Asn | 1 | 0.00 | 0.00 | - | - |
| Ala574Val | 1 | 0.00 | 0.00 | -0.672 | 37.811 |
| Asp695Ala | 1 | 0.00 | 1.00 | 1.655 | 48.128 |
| Gly307Arg | 1 | 1.00 | 0.00 | -0.395 | 20.350 |
| Thr180Lys | 1 | 0.00 | 0.00 | -0.580 | 19.544 |
| Trp477* | 1 | 0.00 | 1.00 | - | - |
| Leu587Pro | 1 | 1.00 | 0.00 | -1.021 | 30.651 |
| Trp351* | 1 | 0.00 | 1.00 | - | - |
| Asp259Glu | 1 | 0.00 | 0.00 | -0.679 | 16.389 |
| Gln36* | 1 | 0.00 | 1.00 | - | - |
| Thr326Met | 1 | 1.00 | 0.00 | 0.099 | 15.246 |
| Gln352* | 1 | 0.00 | 1.00 | - | - |
| Ser140Gly | 1 | 0.00 | 0.00 | -1.084 | 12.600 |
| Trp300* | 1 | 1.00 | 0.00 | - | - |

**Table S6**

**Proportions of co-occurring compensatory mutations with *rpoB* rifampicin resistance mutations.**

| *rpoB* mutation | Frequency | Proportion co-occurring with a compensatory mutation |
| --- | --- | --- |
| Ser450Leu | 4970 | 0.245 |
| Leu430Pro | 140 | 0.036 |
| Ser450Trp | 130 | 0.008 |
| Val170Phe | 73 | 0.164 |
| Gln432Pro | 31 | 0.419 |
| Gln432Lys | 26 | 0.192 |
| Gln432Leu | 21 | 0.143 |
| Pro454His | 7 | 0.143 |

## SUPPLEMENTARY FIGURES

**Figure S1**

**Analysis strategy and numbers of falling into each category**


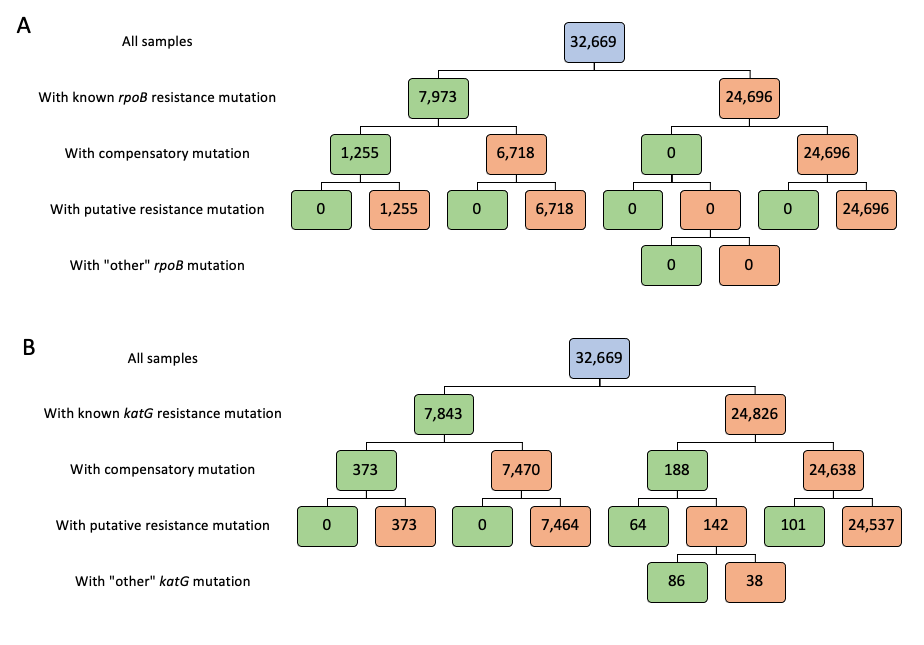


**Figure S2**

**Protein structural model of KatG (7ag8) with known (cyan) and putative (red) mutations highlighted on chain A (blue). The vast majority of putative mutations cluster around the bound heme (green).**


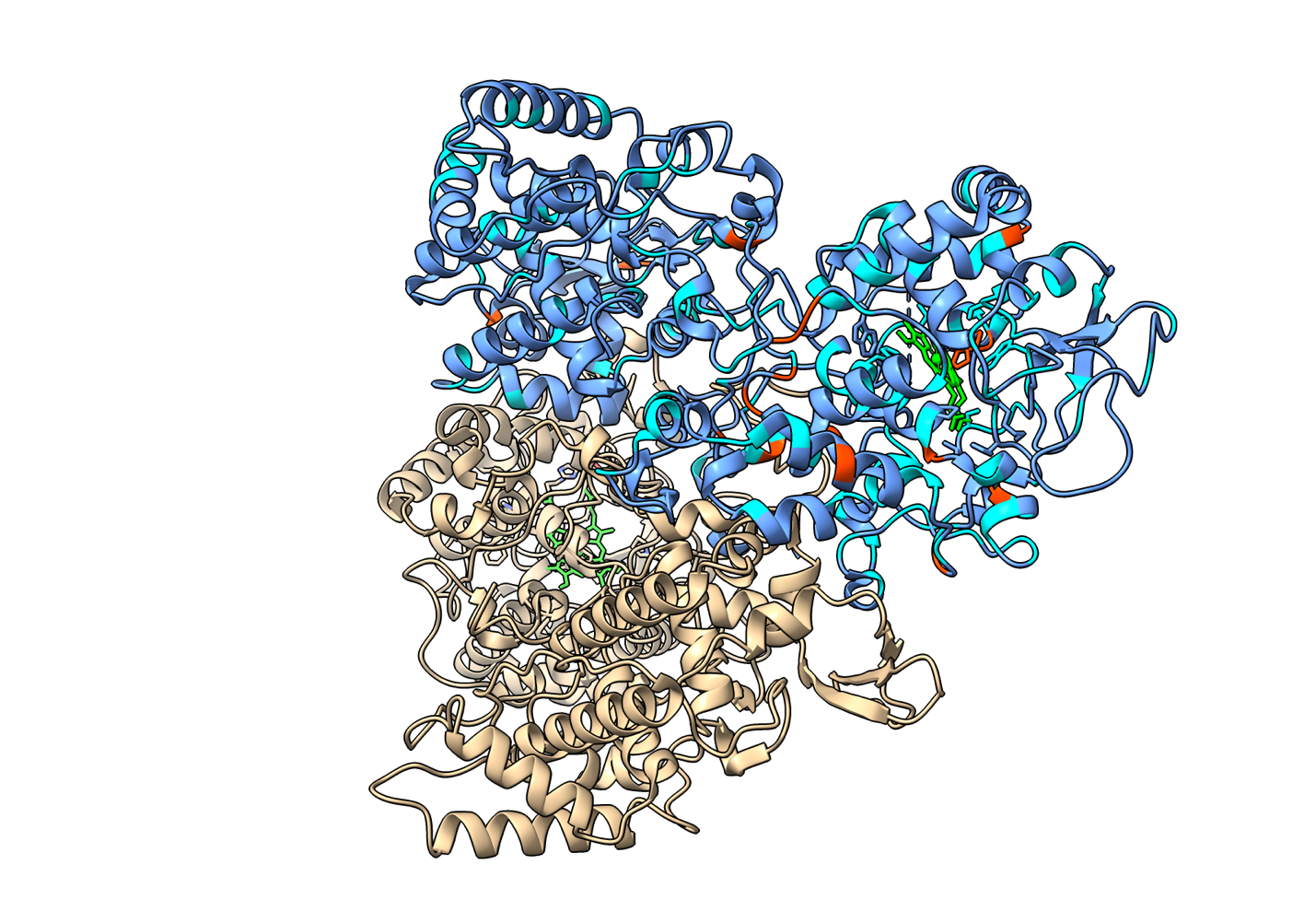

Supplement: Supplementary file 1 — Supplementary Information. [file 41598_2023_27516_MOESM1_ESM.docx]
